# Nonstructural Protein 5A Is Incorporated into Hepatitis C Virus Low-Density Particle through Interaction with Core Protein and Microtubules during Intracellular Transport

**DOI:** 10.1371/journal.pone.0099022

**Published:** 2014-06-06

**Authors:** Chao-Kuen Lai, Vikas Saxena, Chung-Hsin Tseng, King-Song Jeng, Michinori Kohara, Michael M. C. Lai

**Affiliations:** 1 Institute of Molecular Biology, Academia Sinica, Taipei, Taiwan; 2 Department of Molecular Microbiology and Immunology, University of Southern California, Los Angeles, California, United States of America; 3 Department of Microbiology and Immunology, and Center of Infectious Disease and Signaling Research, National Cheng Kung University, Tainan, Taiwan; 4 Graduate Institute of Toxicology, National Taiwan University, Taipei, Taiwan; 5 Department of Microbiology and Cell Biology, Tokyo Metropolitan Institute of Medical Science, Tokyo, Japan; Saint Louis University, United States of America

## Abstract

Nonstructural protein 5A (NS5A) of hepatitis C virus (HCV) serves dual functions in viral RNA replication and virus assembly. Here, we demonstrate that HCV replication complex along with NS5A and Core protein was transported to the lipid droplet (LD) through microtubules, and NS5A-Core complexes were then transported from LD through early-to-late endosomes to the plasma membrane via microtubules. Further studies by cofractionation analysis and immunoelectron microscopy of the released particles showed that NS5A-Core complexes, but not NS4B, were present in the low-density fractions, but not in the high-density fractions, of the HCV RNA-containing virions and associated with the internal virion core. Furthermore, exosomal markers CD63 and CD81 were also detected in the low-density fractions, but not in the high-density fractions. Overall, our results suggest that HCV NS5A is associated with the core of the low-density virus particles which exit the cell through a preexisting endosome/exosome pathway and may contribute to HCV natural infection.

## Introduction

Hepatitis C virus (HCV) is a major causative agent of chronic hepatitis, liver cirrhosis, and hepatocellular carcinoma. HCV is an enveloped virus with a 9.6-kb positive-strand RNA genome. This genome encodes a large polyprotein, which is processed by host and viral proteases into 10 viral proteins that consist of three structural proteins, six nonstructural proteins, and a small hydrophobic peptide, p7 [1], [2]. The structural proteins, Core protein and two envelope glycoproteins E1 and E2, are derived from the N terminal portion of the polyprotein and constitute physical virion components. The nonstructural (NS) proteins, NS2, NS3, NS4A, NS4B, NS5A, and NS5B, are derived from the C terminal portion of the polyprotein. Most of the NS proteins (with the exception of NS2) are involved in HCV replication [3], [4]. HCV RNA is synthesized in the replication complex (RC), which exists in the membranous web derived from altered ER membranes [5], [6]. The HCV RC is transported on microtubules and this transport is facilitated by the interaction of NS3 and NS5A with tubulin [7]. The intact microtubule network also is directly involved in HCV RNA replication [8]–[10] and virus release [10], [11].

Following HCV RNA replication, Core protein and NS5A serve as central regulators of virus assembly [12]. Core protein forms multimers [13] and interacts with the viral RNA [14] to form the viral nucleocapsid. The Core protein is localized mainly on the surface of the lipid droplets (LDs) [15], [16], which is essential for the production of infectious HCV particles [15]. Further, Core protein promotes the accumulation of LDs to facilitate virus assembly [11], [17] and recruits viral RCs to LD-associated membranes [15]. Thereby, viral RNA interacts with Core protein in juxtaposition to LD for virus packaging. Moreover, the interaction between NS5A and Core protein is essential for the recruitment of the viral RCs to LDs and plays an important role in virus assembly [18], [19]. However, how viral RCs and Core protein target to LD remains unclear. In addition to NS5A, other NS proteins, including NS2, NS3, and NS4B, have also been shown to influence the production of infectious virus [12]. Up to now, it is not known whether the NS proteins are incorporated into infectious virions.

Previous studies have indicated that cell culture- [20]–[24] and patients' serum-derived [25]–[29] HCV particles display heterogeneous diameters (from 35 to 145 nm) and have a broad range of buoyant density (between 1.01 g/ml and 1.17 g/ml). The main peak of both viral Core protein and RNA exhibited at a density of 1.15 to 1.17 g/ml in the cell culture derived-HCV (HCVcc) [30], [31], and the highest specific infectivity of extracellular virion was observed at a density of 1.14 g/ml [20]. Notably, the low-density fraction (density of <1.1 g/ml) displays exosome-like structures and also contains infectivity [20], but the nature and origin of their properties are still unknown.

Many types of cell continuously secrete a large number of microvesicles, called exosomes, which have a diameter of approximately 50–150 nm and have a buoyant density between 1.08 g/ml and 1.22 g/ml [32]. Exosomes are released into the extracellular space from late endosomes/multivesicular bodies (MVBs) fusion with the plasma membrane [33]. More recently, the exosomes derived from cells containing HCV subgenomic replicon have been demonstrated to contain HCV RNA, but not viral NS proteins [34]. Our previous results [10] have shown that HCV Core proteins are transported from early to late endosomes/MVB in HCV-infected cells. However, it is not known whether any HCV proteins are incorporated into the released exosomes from HCV-infected cells.

In this study, the trafficking mechanism of the NS5A and Core proteins is defined further. Both NS5A and Core proteins are found to be closely associated with and co-transported along the microtubules from the perinuclear region of cells via the LDs and endosomes to the plasma membrane. This association of NS5A-Core proteins implicated them in virus assembly as well as release. Interestingly, we found that both NS5A and Core, in addition to exosomal proteins CD63 and CD81, were detected in the low-density HCV particles (1.083 to 1.098 g/ml) with low-grade infectivity. NS5A appeared to be incorporated into HCV particles through interaction with Core protein and microtubules during intracellular transport. Our data suggest that NS5A-containing, low-density HCV particles were released in the form of exosome.

## Materials and Methods

### Cells and plasmid

Huh7.5 cells, a mutant line of Huh7 cells that support HCV replication at high efficiency [35] were cultured in Dulbecco's Modified Eagle's Medium (DMEM) containing either 10% fetal bovine serum (FBS) or 10% dialyzed FBS [36] for a general subculturing and for preparation of purified HCV, respectively. Rep 1.1 cells [7], [37] are Huh7 cells that harbor a genotype 1b HCV subgenomic replicon. They were grown in the afore-mentioned medium containing 0.5 mg/ml of G418. Plasmid pUC-Jc1, which encodes a chimera genome of HCV J6CF/JFH1, was constructed as previously described [38].

### Antibodies and reagents

Antibodies used in this study included anti-NS5A (Austral Biologicals), anti-Core (Affinity Bioreagents Inc.), anti-bromodeoxyuridine (Sigma-Aldrich), anti-EEA1 or -CD63 or -LAMP-1 (Santa Cruz Biotechnology), mouse anti-CD81 (BD Pharmingen), rabbit anti-CD81 (Santa Cruz Biotechnology), anti-E2 (GeneTex, Inc.), anti-calnexin (Assay designs/Stressgen), and anti-mouse or -rabbit colloidal gold conjugates (Jackson ImmunoResearch Inc.). Rabbit polyclonal Abs against Core (RR8) and NS4B (RR12) were described previously [15]. Cy3-conjugated primary Ab to β-tubulin and nonspecific normal mouse immunoglobulin G (IgG) were obtained from Sigma-Aldrich. Secondary Abs against mouse, rabbit and goat were purchased from Invitrogen Molecular Probes.

Reagents used were Nocodazole (Sigma-Aldrich), taxol (paclitaxel; Sigma-Aldrich), BODIPY 493/503 (Invitrogen Molecular Probes), 4',6-Diamidino-2-phenylindole (DAPI; Invitrogen Molecular Probes) and wheatgerm agglutinin (WGA) Alexa Fluor 647 conjugate (Invitrogen Molecular Probes).

### Preparation of purified HCV particles

 The Jc1 viruses were produced and titrated in Huh7.5 cells based on a previously described method [7]. The same batch of culture supernatant from virus-infected and the control cells, including HCV subgenomic replicon cells (HCV assembly-defective replicon cells) and uninfected cells, was used in all experiments. The concentrated HCV Jc1 and controls were subjected to 10 to 50% sucrose gradient sedimentation centrifugation as previously described [39]. A total of 15 fractions of 0.75 ml each was collected from the bottom to the top of the sucrose gradient and monitored for HCV RNA by using quantitative PCR (qPCR) [10]. Fractions containing the HCV RNA signal (fractions 7 to 8 and fractions 11 to 13) and their uninfected counterparts were pooled and dialyzed against TNE buffer (10 mM Tris, 150 mM NaCl, 2 mM ethylene diamine tetraacetic acid) overnight at 4°C. The virus-containing fractions were then concentrated by 10-fold in Ultracel-3k concentration devices (Millipore) and further processed for electron microscopy applications or infection. Infectivity of each fraction was determined by quantifying the amounts of intracellular HCV RNA levels at day 3 postinfection (p.i.).

### Determination of HCV infectivity by quantitative reverse transcription-PCR (qRT-PCR) of intracellular HCV RNA of infected cells

Huh7.5 cells in six-well plates were infected with concentrated HCV from each fraction of the sucrose gradient suspended in DMEM at 37°C for 3 h. Cells were washed with PBS and incubated with 2 ml of DMEM containing 10% FBS. At 3 days postinfection, total RNA was isolated from cell lysates using a High Pure RNA isolating kit (Roche). Viral RNA was isolated from cell culture supernatants using a QIAamp viral RNA kit (Qiagen). qRT-PCR was performed as described previously [10], [40].

### Electron microscopy (EM)

 The procedure of EM was carried out exactly as described previously [40]. For immuno-EM, samples were first incubated with an anti-NS5A, anti-Core or anti-E2 mouse MAb, and followed by incubation with colloidal gold particles of 18 nm conjugated to anti-mouse immunoglobulin G. For immuno-EM of the purified viruses, two microliters of purified virus were adsorbed onto carbon-coated grids. Grids were fixed with 2% paraformaldehyde for 10 min and blocked in a solution of 0.5% bovine serum albumin (BSA) for 20 min. The grids were incubated with primary Abs and normal mouse IgG for overnight at 4°C. Grids were then washed and incubated with goat anti-mouse and/or goat anti-rabbit colloidal gold particles (6- and 12-nm diameter) for 1 h at room temperature. After extensive washing, the grids were stained with 1% uranyl acetate for 1 min. For analysis of HCV core particle, the purified virus was treated with 0.01% saponin in PBS for 20 min before processing. All samples were analyzed under a Tecnai Spirit transmission electron microscope (FEI Co) at 120 kV.

### Labeling of *de novo*-synthesized viral RNA

Cell labeling with 5-bromouridine 5'-triphosphate (BrUTP) was performed according to the methods described previously [7]. HCV-infected cells (at day 10 p.i.) were grown on 4-well chamber slides. One day after seeding, cells were incubated with actinomycin D (10 µg/ml) for 30 min. Then, 20 µl of BrUTP/Fugene 6 (Roche Molecular Biochemicals) mixture were added to each well containing 500 µl medium with actinomycin D (10 µg/ml). After 30 min of incubation at 37°C, cells were treated with 10 µM nocodazole or taxol for 1 h, and then fixed and processed for immunofluorescence staining as described below.

### Immunofluorescence staining

Cells were gown on glass chamber slides. Cell fixation and immunostaining were performed by the methods described by Listenberger L. L. and Brown D. A. [41]. Photographs of the cells were taken with a confocal microscope (Zeiss Confocal Laser Scanning Microscope LSM 510META-NLO).

### Image processing and quantitative analysis

The procedure for quantitative colocalization analysis used in this study followed the published method [10]. The weighted colocalization coefficient is the sum of intensities of colocalization pixels relative to the overall sum of pixel intensities above the threshold. It was used to determine the relative levels of colocalization between Core protein, NS5A or NS4B with the protein of interest (e.g., calnexin, LD). The shortest and longest average distances between the BrUTP-labeled viral RNA signal center and the nearest edge of LD were calculated manually using ZEN software (Zen 2009 light edition; Carl Zeiss Inc). The number of BrUTP-labeled viral RNA and antibody-labeled signals of viral proteins at the plasma membrane was manually counted in an original magnification of 630. Total fluorescence intensity values of Core protein and NS5A for individual cells were measured using the MetaMorph software (Universal Imaging Corporation), using 8 bit images. The gray-scale of the 8-bit images ranged from 0 (black) to 256 (white). Image analysis was carried out using the Integrated Morphometry Analysis program provided by MetaMorph. Fluorescence intensity is expressed as the integrated value of all pixels per cell that exceed the inclusive threshold value set at 40. A total of 20 cells were used in each experimental condition from two independent experiments.

### MTS assay

A CellTiter96A_Queous_ One solution cell proliferation assay kit (Promega) was used to evaluate cell viability, which was performed as described previously [40].

## Results

### HCV NS5A colocalizes with Core protein on lipid droplet, and the complex is closely associated with microtubules

**Figure 1:**
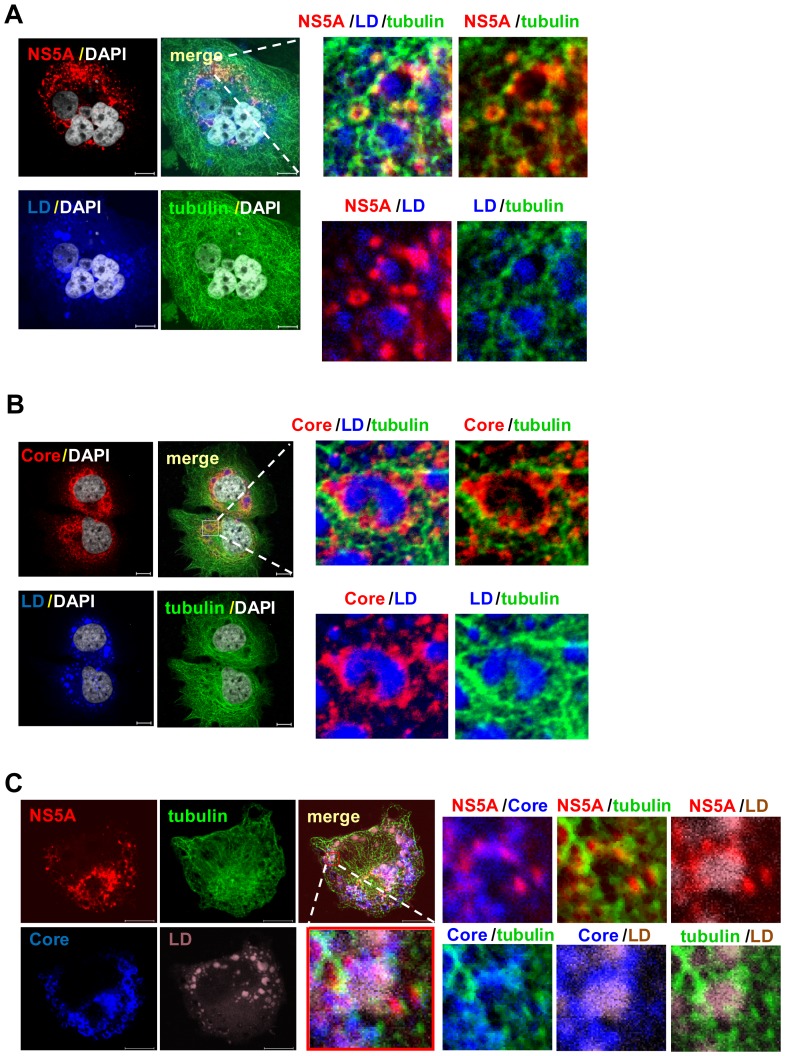
Colocalization of NS5A with Core protein and microtubules in lipid droplets. HCV-infected cells (at day 10 p.i.) were co-stained with anti-tubulin (green), -NS5A (red) (A) and/or -Core (red, B; blue, C) antibodies. Lipid droplets (LDs) were stained with BODYPI 493/503 (blue, A and B; brown, C) and nuclei with DAPI (gray). Stained cells were examined by confocal fluorescence microscope. Merging of the images in the green, red, blue, and gray channels generated the pictures in Fig. A and B. Fig. C was generated by merging the images in the green, red, blue, and brown channels. Yellow indicates overlapping localization of the green and red channels, cyan indicates overlapping localization of the green and blue channels, magenta indicates overlapping localization of the red and blue channels, and white indicates overlapping localization of the red, green, and blue channels. The upper third panels in Fig. A and B and the lower third panel in Fig. C are enlarged areas from the merged image. The same enlarged area is defined in terms of two proteins at a time, as indicated, in the adjoining three panels in Fig. A and B, and six panels in Fig. C. Bars, 10 µm.

Our previous studies have shown that microtubule provides the track for the movement of HCV RCs through NS5A-microtubule interaction [7] and that this transport is required for virus release [10]. We propose that microtubules also provide tracks for the transport of NS5A or the NS5A-containing RC and Core protein to reach the LD, where virus assembly occurs. To test this possibility, we first investigated whether Core-containing LDs colocalized with NS5A and associated with microtubules in HCV-infected Huh7.5 cells [at day 10 postinfection (p.i.)]. Immunofluorescence staining revealed that NS5A and Core protein together (Fig. 1C), or either one alone (Fig. 1A and 1B), is colocalized on the surface of LD and is closely associated with tubulins.

### The microtubule network is required for the trafficking of NS5A or the NS5A-containing replication complexes and Core protein to the lipid droplet

**Figure 2:**
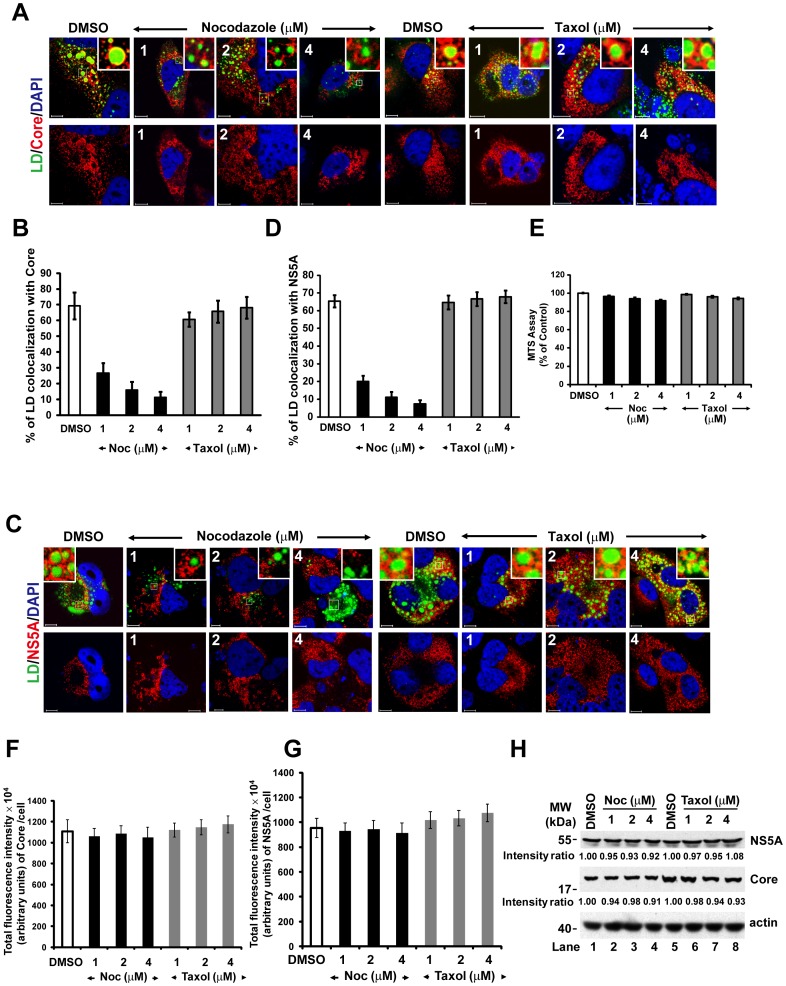
Nocodazole blocks the transport of NS5A and Core protein to the lipid droplet in a dose-dependent manner. Huh7.5 cells were first infected with HCV Jc1 virus at a multiplicity of infection of 0.5 for 3 h, and then the cells were treated with either nocodazole (1 to 4 µM) or taxol (1 to 4 µM) at various concentrations for 2 days. The cells were stained with anti-Core (red) (A) or -NS5A (red) (C) antibodies. LDs and nuclei were stained with BODYPI 493/503 (green) and DAPI (blue), respectively. Enlarged views of parts of every image are shown (insets). The same images are shown for Core (red) or NS5A (red) and with DAPI (blue) in the lower panels of Fig. A and C, respectively. Colocalization efficiency between LD and Core protein (B) and between LD and NS5A (D) was analyzed by using Zeiss LSM Zen software. (E) Analysis of cellular proliferation and survival by MTS assay. (F, G) Quantitation of confocal microscopic fluorescent signals of Core and NS5A in cells. The total fluorescence intensities of Core protein and NS5A were measured using MetaMorph Integrated Morphometry Analysis. A total of 20 cells were used for calculation of colocalization efficiency and total fluorescence intensity from two independent experiments and error bars represent standard deviations of the mean. Noc, nocodazole. Bars, 10 µm. (H) In parallel, the cell lysates were collected and then immunoblotted with antibodies against Core and NS5A. Results were quantified by PhosphorImager counting.

We used nocodazole (which induces microtubule depolymerization) or taxol (which stabilizes tubulin polymerization) to examine whether intact microtubules are required for the transport of Core protein, NS5A or viral RCs to the LD. Huh7.5 cells were first infected with HCV, and treated with either nocodazole or taxol at 3 hr p.i. for 2 days. The colocalization coefficient of LD with Core protein or NS5A was then calculated. Under these conditions, cell viability was not affected, as revealed by 3-(4,5-dimethylthiazol-2-yl)-5-(3-carboxymethoxyphenyl)-2-(4-sulfophenyl)-2H-tetrazolium salt (MTS) assay (Fig. 2E).

**Figure 3:**
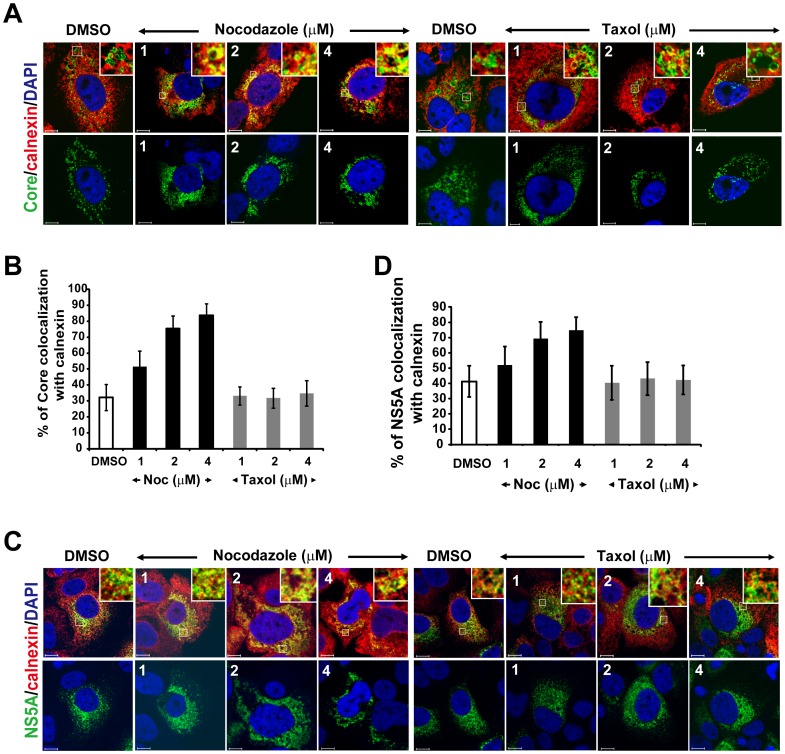
Effects of Nocodazole on movement of NS5A and Core protein. Huh7.5 cells were first infected with HCV Jc1 virus at a multiplicity of infection of 0.5 for 3 h, and then the cells were treated with either nocodazole (1 to 4 µM) or taxol (1 to 4 µM) at various concentrations for 2 days for the analysis of colocalization of Core protein or NS5A and calnexin. The cells were co-stained with a polyclonal antibody against calnexin (red) to visualize the ER and a MAb against Core protein (green) (A) or NS5A (green) (C). Nuclei were stained with DAPI (blue). Enlarged views of parts of every image are shown (insets). The same images are shown for Core (green) or NS5A (green) and with DAPI (blue) in the lower panels of Fig. A and C, respectively. Colocalization efficiency between Core protein and calnexin (B) and between NS5A and calnexin (D) was analyzed by using Zeiss LSM Zen software. A total of 20 cells were used for calculation of colocalization efficiency from two independent experiments and error bars represent standard deviations of the mean.

Under the control conditions [dimethyl sulfoxide (DMSO)], LD colocalized with Core protein throughout the entire cytoplasm, including the perinuclear region; the proportion of LD that colocalized with Core protein was 69%. When the cells were treated with increasing concentrations of nocodazole, a dose-dependent decrease in the colocalization coefficient of LD with Core protein was observed (Fig. 2A upper panels and 2B). The colocalization coefficient of LD with NS5A also decreased correspondingly (Fig. 2C upper panels and 2D). Taxol did not have effects on this coefficient. In order to rule out the possibility that the dose-dependent decrease in the colocalization of LD with Core or NS5A by nocodazole treatments might have been the consequence of a decrease in the amounts of Core and NS5A proteins, a further analysis of Core protein (Fig. 2A and 3A, lower panels) and NS5A (Fig. 2C and 3C, lower panels) labeling in cells was carried out using the MetaMorph Integrated Morphometry Analysis. In nocodazole-treated cells, there was no significant change on the total fluorescence intensities of Core and NS5A relative to control cells (Fig. 2F and 2G). Immunoblot analysis also showed that the levels of NS5A and Core proteins in the cells were not significantly affected by the low concentration (up to 4 µM) of nocodazole or taxol used (Fig. 2H,). This was in contrast to the effects of nocodazole at 10 or more µM used in most of the published studies, which inhibited HCV RNA replication and thereby HCV protein level [8], [10], [42]. These data support the conclusion that the nocodazole treatment at low concentrations affected the colocalization of Core and NS5A with LD (Fig. 2B and Fig. 2D). Taken together, these results indicated that microtubules are involved in the transport of Core protein and NS5A to the LD.

Newly synthesized membrane proteins generally leave the endoplasmic reticulum (ER) and transported to other destinations in the cells. Therefore, we next investigated whether NS5A and Core proteins are transported from ER to LDs along the microtubules. In the presence of DMSO or taxol, Core protein was colocalized with the ER marker protein calnexin throughout the entire cytoplasm, including the perinuclear region, with a colocalization coefficient of 32% (Fig. 3A and 3B). In contrast, the nocodazole treatment caused a dose-dependent increase of colocalization coefficient to 75% and 84% at 2 and 4 µM nocodazole, respectively (Fig. 3A, upper panels and 3B). Similarly, the colocalization coefficient of NS5A with calnexin increased from 41% to 69% and 75%, respectively (Fig. 3C, upper panels and 3D). Thus, the nocodazole treatment caused an increased accumulation of Core protein and NS5A in the ER and corresponding decrease in the LDs. Taken together, these results suggested that NS5A and Core protein are translocated from ER to LDs in a microtubule-dependent manner.

**Figure 4:**
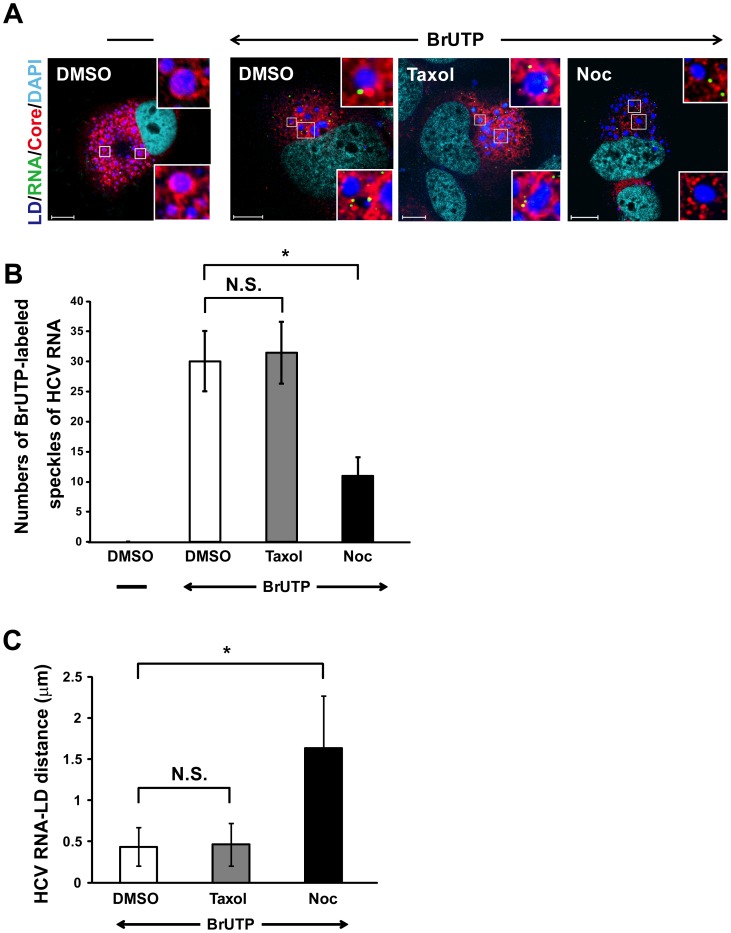
Nocodazole affects the movement of viral replication complexes (RCs) to the LDs. (A) The HCV-infected cells (at day 10 p.i.) were transfected with BrUTP in the presence of actinomycin D for 1 h and then treated with either 10 µM nocodazole or 10 µM taxol for 1 h. The cells were co-stained with mouse MAb against bromodeoxyuridine (green) and rabbit polyclonal antibodies against Core (RR8) (red). LDs and nuclei were stained with BODYPI 493/503 (blue) and DAPI (cyan), respectively. Enlarged views of parts of every image are shown (insets). (B) The number of BrUTP-labeled viral RNA was counted manually using an original magnification of ×630 and followed by a quantitation analysis performed by an observer blinded to the experimental treatment. (C) The average distance between the center of the signal emitted by the BrUTP-labeled viral RNA and the nearest edge of LD (HCV RNA-LD distance) were analyzed by using Zeiss LSM Zen software. A total of 20 cells were used for quantitation and calculation of the HCV RNA-LD distance and the number of BrUTP-labeled viral RNA from two independent experiments and error bars represent standard deviations of the mean. N.S., non-significance; *, P<0.01; Noc, nocodazole. Bars, 10 µm.

We further confirmed the mode of transportation of the HCV RC to the LD by immunofluorescent labeling of newly synthesized HCV RNA with BrUTP [43]. In the DMSO control and taxol-treated cells, the number of BrUTP-labeled speckles averaged 30 per cell, and the average distance between speckle and LD was 0.43 µm (Fig. 4). In the presence of nocodazole, the number of speckles was reduced to an average of 11 per cell (Fig. 4B), indicating that the HCV RNA replication is partially suppressed by nocodazole treatments, consistent with the previous reports [8]–[10]. Correspondingly, the average distance between the speckle center and the nearest edge of LD increased from 0.43 µm in the DMSO controls and taxol-treated cells to 1.63 µm in the nocodazole-treated cells (Fig. 4C), suggesting that the HCV RCs are normally delivered to the vicinity of the LDs through microtubule and that this transport is disrupted by nocodazole. Taken together, these data suggest that NS5A or the NS5A-containing replication complexes and Core protein are transported to the LDs through microtubules.

### NS5A and Core protein complex is cotransported from the perinuclear region to the plasma membrane via microtubule

**Figure 5:**
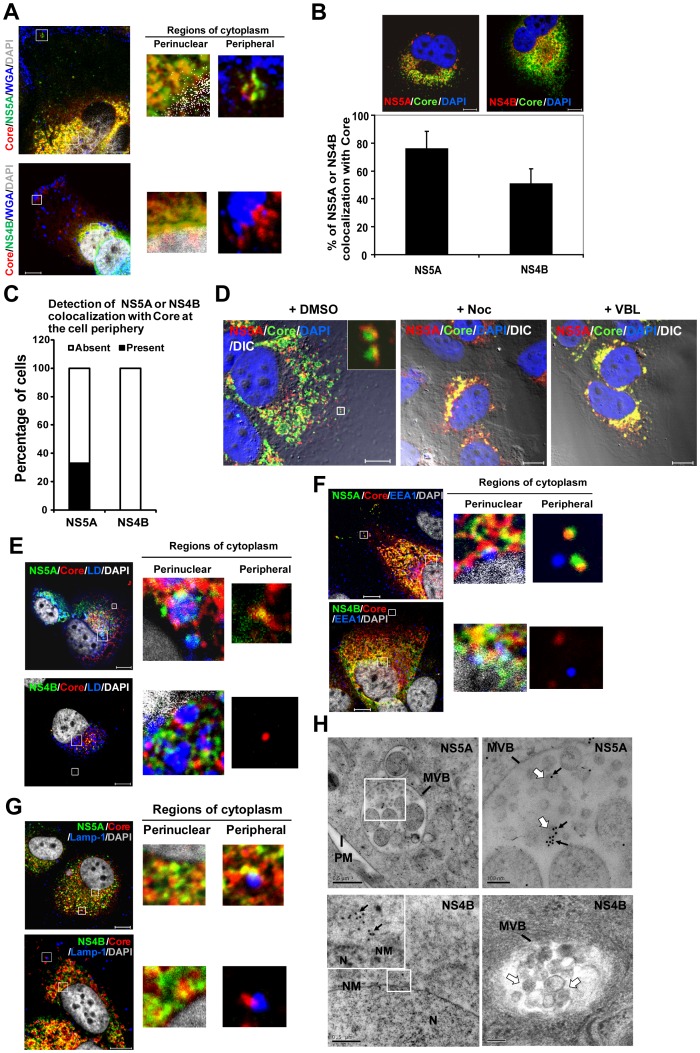
Core-NS5A complexes are transported from perinuclear region to early and late endosomes. (A) The HCV-infected cells (at day 10 p.i.) were labeled with antibodies specific for Core protein (red) and NS5A (green) (upper row) or NS4B (green) (lower row). Plasma membrane and nuclei were stained with WGA Alexa Fluor 647 conjugate (blue) and DAPI (gray), respectively. In parallel with panel A, the HCV-infected cells were labeled with antibodies to Core protein (green) and NS5A (red) or NS4B (red) (B, upper rows). Nuclei were stained with DAPI (blue). Colocalization efficiency between Core protein and NS5A or NS4B was analyzed by using Zeiss LSM Zen software (B, lower panel). Error bars represent standard deviations of the mean from 20 cells in two independent experiments. The images were analyzed by using MetaMorph, and the proportion of cells (of 200 counted) in which the NS5A or NS4B colocalized with Core protein at the cell periphery was calculated (C). (D) Effects of nocodazole (Noc) and vinblastine (VBL) on movement of Core-NS5A protein complex to the cell periphery. The HCV-infected cells (at day 10 p.i.) were treated with nocodazole, vinblastine, or DMSO for 3 h. The cells were labeled with antibodies to Core protein (green), NS5A (red). Nuclei were stained with DAPI (blue). Images with differential interference contrast (DIC) and fluorescence images were then merged. An enlarged view of part of image is shown (inset). In parallel with panel A, the HCV-infected cells were labeled with antibodies to Core protein (red) (E, F, G), NS5A (green, E, F, G) (upper rows), NS4B (green, E, F, G) (lower rows), EEA1 (blue) (F) and Lamp-1 (blue) (G). LDs and nuclei were stained with BODYPI 493/503 (blue) (E) and DAPI (gray), respectively. The second and third panels in each row are magnified views, marked with a white box in the panel at the extreme left, of the perinuclear and the peripheral regions of cytoplasm, respectively (A, E, F, G). Colocalization of Core protein with NS5A or NS4B is depicted as yellow (A, B, D, E, F, G). Colocalization of Core-NS5A or -NS4B complexes with LDs (E), early endosomes (F) or late endosomes (G) is depicted as white. Bars, 10 µm. (H) In parallel, the HCV-infected cells was labeled with anti-NS5A or -NS4B Ab. Bound antibodies were detected using anti-mouse or -rabbit secondary antibodies conjugated to 18- or 12-nm gold particles, respectively. Sections were visualized by EM. Arrows, gold-labeled NS5A (18 nm) and NS4B (12 nm) are indicated. MVB contains several intravesicular vesicles (white arrows). An enlarged view of the perinuclear region of lower left image is shown (inset). Arrows, gold-labeled NS5A and NS4B. PM, plasma membrane; MVB, multivesicular bodies; N, nucleus; NM, nuclear membrane; Bars, 500 nm (left panels) and 100 nm (right panels).

NS5A, together with Core protein, is involved in virus assembly; the assembled virions are presumably transported to the plasma membrane to be released off the cell. Therefore, we next analyzed whether NS5A is involved in the post-assembly processes of viral life cycle. For this purpose, we examined the colocalization of NS5A with Core protein in two regions of the cytoplasm, *viz*. the perinuclear (region just around the nucleus) and the peripheral (region just underneath the plasma membrane) regions. HCV-infected cells were co-stained with fluorescent (Alexa 647) WGA (which binds glycoproteins on the cell membrane), which serves as a membrane marker, anti-Core, and either anti-NS5A or -NS4B Abs. As shown in Fig. 5A, NS5A and Core protein colocalized both in the perinuclear region and at the cell periphery. In contrast, NS4B colocalized with the Core protein only in the perinuclear region of cytoplasm, but not in the peripheral region. Analysis of a large number of cells indicated that Core protein was colocalized with NS5A throughout the entire cytoplasm, but not with NS4B (Fig. 5B, upper panels). The total fraction of NS5A that colocalized with Core protein was 76%, while the corresponding NS4B was 51% (Fig. 5B, lower panel). Further, the NS5A-Core complex colocalized in the peripheral region in 33% of the cells, whereas NS4B-Core did not colocalize at all in the same region (Fig. 5C). We next examined the possibility that the Core-NS5A complexes are also transported along microtubule to the cell periphery. In the DMSO-treated cells, NS5A colocalized with Core protein in both the perinuclear and peripheral regions (Fig.5D). After treatment with either nocodazole or vinblastine, the Core-NS5A complexes clustered almost exclusively in the perinuclear region (Fig. 5D). These results suggested that microtubules are required for the transport of Core-NS5A complexes to the cell periphery. This observation further suggests that NS5A, but not NS4B, is involved in the post-assembly transport of virus particles.

To further evaluate the involvement of the NS5A-Core complex in HCV assembly and release, we investigated the localization of this complex with respect to LDs [15], early and late endosomes [10], [44]–[47], and microtubules [10], [11], all of which are involved in the various steps of HCV assembly and exit. The HCV-infected cells were co-stained with the LD marker (BODIPY 493/503), the early endosome marker (early endosome antigen 1; EEA1), the late endosome marker (lysosome-associated membrane protein-1; Lamp-1), or the microtubule marker (β-tubulin). In the perinuclear region of cytoplasm, both the NS5A-Core and NS4B-Core complexes colocalized with the LDs and the early endosomes (Fig. 5E and 5F). In contrast, in the peripheral region, only the NS5A-Core complex, but not the NS4B-Core, colocalized with the late endosomes (Fig. 5G). Immunoelectron microscopy (immuno-EM) further showed that the late endosome/MVB contained NS5A, but not NS4B, the latter being mainly in the perinuclear regions (Fig. 5H). These results suggested strongly that NS5A-Core complexes, but not NS4B, are transported through early-to-late endosomes following virus assembly at LD.

**Figure 6:**
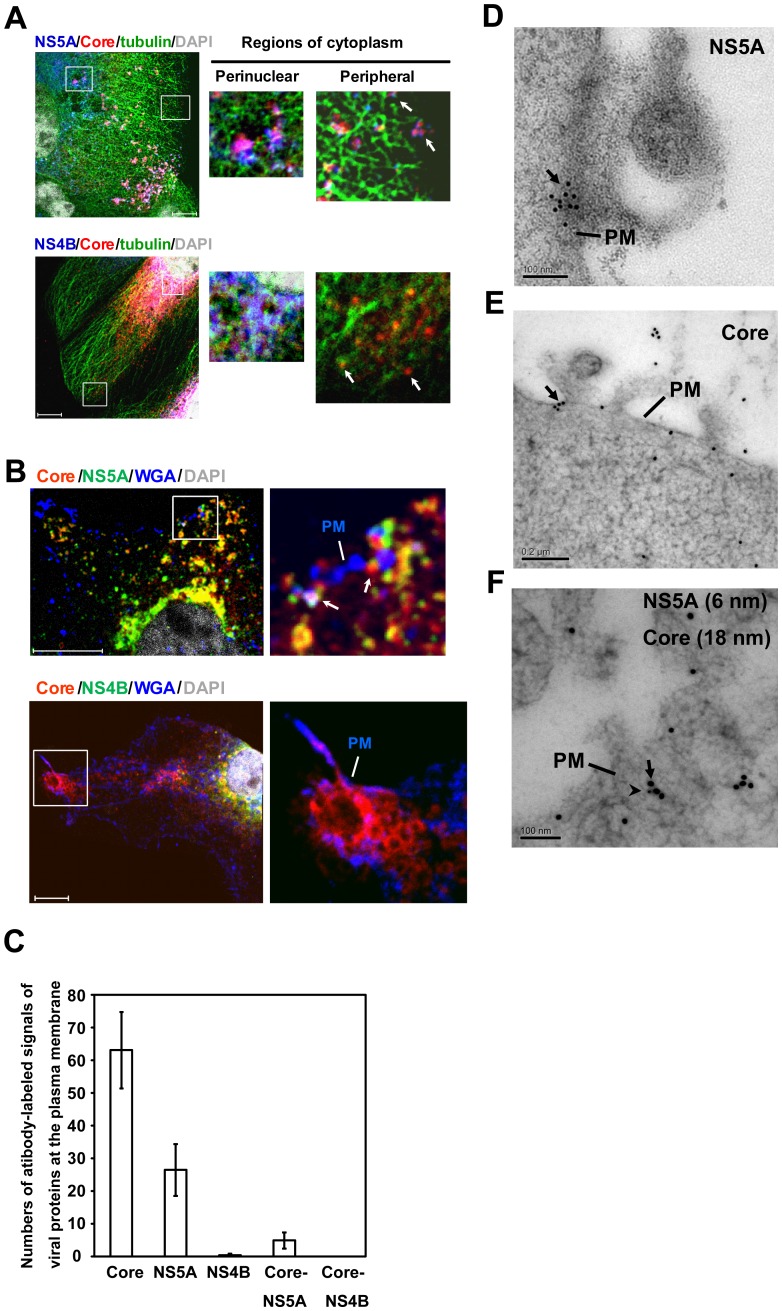
Core-NS5A complexes are transported from perinuclear region to the plasma membrane via microtubules. The HCV-infected cells (at day 10 p.i.) were labeled with antibodies to Core protein (red) (A, B), NS5A (blue, A; green, B) (upper rows), NS4B (blue, A; green, B) (lower row) or tubulin (green) (A). Plasma membrane and nuclei were stained with WGA Alexa Fluor 647 conjugate (blue) (B) and DAPI (gray), respectively. The second and third panels in each row are magnified views, marked with a white box in the panel at the extreme left, of the perinuclear and the peripheral regions of cytoplasm, respectively (A). Colocalization of Core with NS5A or NS4B is depicted as magenta (A) or yellow (B). Colocalization of Core-NS5A or -NS4B protein complexes with microtubules (A) or plasma membrane (B) is depicted as white. The microtubule end (white arrow) is closest to the cell periphery (A). (B) At the right is an enlarged area, marked with a white box, from the merged image. Colocalization of NS5A with Core protein was observed in the membrane curvature (white arrow). PM, plasma membrane; Bars, 10 µm. (C) Quantitation of antibody-labeled signals of viral proteins at the plasma membrane. Images from 20 cells were counted manually using an original magnification of ×630 and followed by a quantitation analysis performed by an observer blinded to the experimental treatment. (D, E) NS5A and Core are localized in clusters or patches on the plasma membrane. In parallel, the HCV-infected cells were labeled with anti-NS5A (D) or anti-Core (E) antibodies. Bound antibodies were detected using anti-mouse secondary antibodies conjugated to 18-nm gold particles. Sections were visualized by EM. Arrows, gold-labeled NS5A (D) or Core protein (E). (F) Immuno-EM of Core-NS5A complex colocalized at the plasma membrane. The HCV-infected cells were co-labeled with antibodies against NS5A (6 nm) and Core (18 nm). Shown is a view of the plasma membrane. Arrowhead, gold-labeled NS5A. Arrow, gold-labeled Core. PM, plasma membrane; Bars, 100 nm (D, F) and 200 nm (E).

Furthermore, NS5A and Core protein, but not NS4B, colocalized with the microtubules in the peripheral region of cytoplasm, especially at the microtubule end that is closest to the cell periphery (Fig. 6A). Finally, we characterized the relationship of NS5A-Core complexes with the plasma membrane. HCV-infected cells were co-stained with fluorescent (Alexa 647) WGA, anti-Core, and anti-NS5A or -NS4B Abs. As shown in Fig. 6B, NS5A and Core protein colocalized at the plasma membrane, particularly at the membrane curvature, which has been reported to be essential for virus exit [48]. Quantitative analysis showed that the number of anti-Core and anti-NS5A antibodies-labeled signals at the plasma membrane averaged 63 and 26 per cell, respectively, whereas that for anti-NS4B was less than 1. Interestingly, some signals containing both NS5A and Core (5 per cell on average) were also detected at the plasma membrane, whereas no NS4B-Core signals were found (Fig. 6C). Furthermore, immuno-EM showed that NS5A (Fig. 6D), Core protein (Fig.6E) and the Core-NS5A complex (Fig. 6F) localized mainly to patches or clusters on the plasma membrane. Taken together, these results again suggest that at least some of NS5A-Core complexes (or the assembled virions) are transported from LD through early-to-late endosomes to the plasma membrane via microtubules.

### Identification of NS5A and exosomal proteins as low-density HCV virion-associated proteins

**Figure 7:**
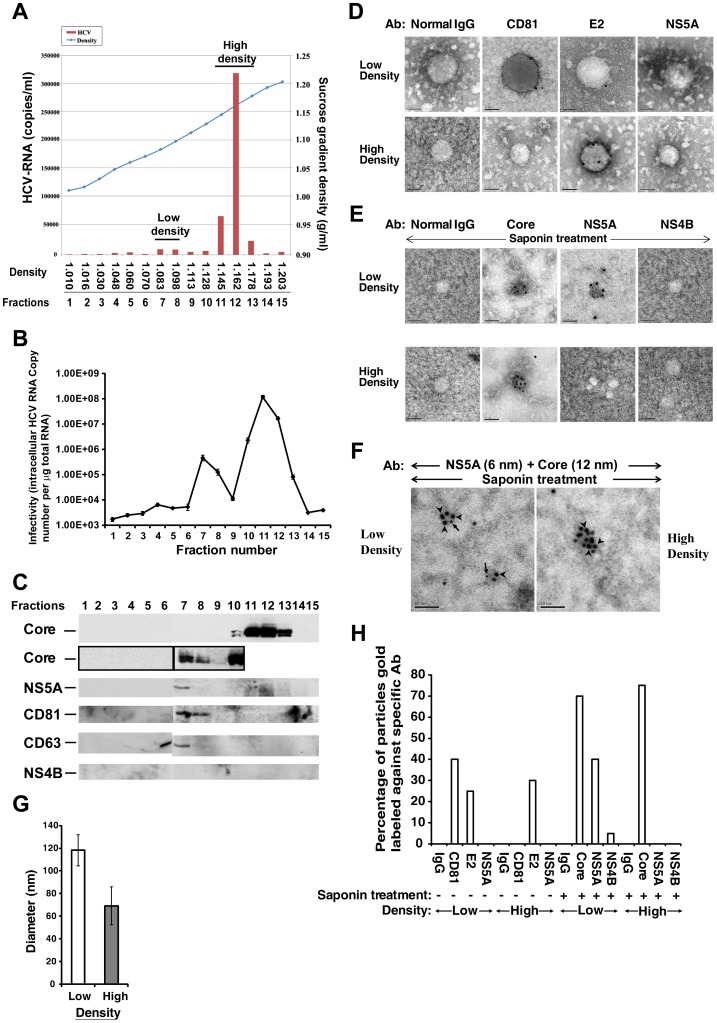
Association of NS5A and exosomal proteins with the low-density HCV particles. HCV virion populations were analyzed by sucrose density gradient ultracentrifugation. (A) Concentrated culture medium collected from HCV Jc1-infected cells was fractionated using a continuous 10–50% sucrose density gradient. HCV RNA levels were determined in each fraction. (B) Infectivity of each density gradient fraction. An aliquot of each fraction was used to infect naive Huh7.5 cells. Intracellular HCV RNA copy number per µg of total RNA was determined 3 days after infection by qRT-PCR. (C) Western blot analysis of each density gradient fraction, shown in panel A, by using antibodies indicated at the left. (D, E and F) Electron micrograph of the viral spherical structures shown by immunogold labeling. HCV particles were purified from two pooled fractions; the low-density particles (from fractions 7 to 8), and the high-density particles (from fractions 11 to 13), after dialysis and concentration. The virus samples were untreated (D) or treated with 0.01% saponin (E, F), as described in Materials and Methods. Grids were incubated with an untreated or saponin-treated purified HCV and then with antibodies against CD81, E2, or NS5A (D) or with antibodies against Core protein, NS5A or NS4B (E), respectively. Bound antibodies were detected using anti-mouse or -rabbit secondary antibodies conjugated to 12-nm gold particles. (F) Core-NS5A complex localized in the virion core of the low-density particles. Grids were incubated with a saponin-treated purified HCV and then co-labeled with antibodies against NS5A (6 nm) and Core (12 nm). To determine antibody specificity, primary antibodies were replaced with nonspecific normal mouse IgG. Bars, 50 nm. (G) The average diameter of the low- (n = 60) and high-density (n = 90) particles was obtained from the combined analysis of two independent viral preparations and error bars represent standard deviations of the mean. (H) Statistics were performed by counting HCV particles and immunogold labeled HCV particles from the combined analysis of two independent viral preparations. Relative proportion of HCV particles that were labeled with antibodies (n = 60).

To confirm that NS5A is released from the plasma membrane as a component of some virion particles, we cultured HCV-infected Huh7.5 cells with dialyzed serum to reduce nonspecific binding of irrelevant proteins to virus particles. The culture supernatant was subject to sucrose gradient sedimentation, and the distribution of the viral RNA and infectivity of HCV were determined. Two peaks, one from fractions 7–8, with density of 1.083 to 1.098 g/ml sucrose (low-density fractions; LDF), and another from fractions 11–13 with density of 1.145 to 1.178 g/ml sucrose (high-density fractions; HDF), were found to contain distinct HCV RNA signals (Fig. 7A). Fraction 12, at 1.162 g/ml sucrose, contains the largest amount of viral RNA, consistent with the previously reported density of free HCV virions [27], [31]. Each fraction was further analyzed for its infectivity on naive Huh7.5 cells. The results showed that both the LDF and HDF from culture supernatant contained infectivity (Fig. 7B), with HDF having approximately 153-fold higher specific infectivity. Thus, both the LDF and HDF are infectious, but LDF contained only 0.65% of the total infectivity; thus, the possibility that the apparent infectivity of LDF may have come from contaminations of HDF cannot be ruled out.

Further, we analyzed the various fractions for the possible presence of Core and NS5A. As shown in Fig. 7C, the HDF contained abundant Core protein, but no NS5A or NS4B. In contrast, the low-density fraction 7 (1.083 g/ml) contained NS5A in addition to Core protein, but no NS4B. Recent studies have revealed that HCV virion release requires late endosome (or multivesicular body; MVB) [10], the functional endosomal sorting complex required for transport (ESCRT) [46], [47] and hepatocyte receptor tyrosine kinase substrate (Hrs) [45], all of which are involved in the biogenesis of MVB and exosome secretion. In addition, HCV particles are associated with circulating exosomes in hepatitis patients' serum [49]. We therefore investigated whether HCV virion release from cell membrane occurs through the exosome pathway. We probed each sucrose density fraction for the presence of two exosomal markers *viz.* CD81 and CD63. Interestingly, both CD81 and CD63 were detected in LDF (Fig. 7C), but not HDF, suggesting that the exosome is involved only in LDF virus release.

Since exosomes are derived from endosomes and are also secreted from normal cells to participate in a variety of cellular functions [50], we also analyzed the sucrose gradient fractions of extracellular medium obtained from naïve Huh7.5 cells and cells harboring an HCV subgenomic replicon. None of the fractions obtained from these cells contained HCV NS5A protein (data not shown), consistent with the previous report [34]. These data suggest that LDF contained HCV particles released through exosome-like structure, and contained NS5A.

The morphology and composition of virus particles in both the LDF and HDF were further characterized. The diameter of particles ranged from 105 to 132 nm (n = 60) and 52 to 86 nm (n = 90) in the low- and high-density pools, respectively (Fig. 7D and 7G). The size heterogeneity of HCV particles has been described previously [20], [25], [31]. Correspondingly, particles of ∼130 nm and ∼70 nm in diameter, probably representing the low- and high-density virus particles, respectively, were observed in the extracellular space surrounding the HCV-infected cells (Fig. S1). After treatment with saponin (0.01%), which removed the viral envelope, the size of the particles was reduced to 35-41 nm for both the LDF and HDF viruses (Fig. 7E and 7F), consistent with its being the internal viral core [20], [51].

Next, the purified virions were characterized for the presence of viral proteins and exosomal marker CD81 by immuno-EM. The particles in the low-density pool could be stained with anti-CD81 and -E2 MAbs, but not with a purified normal IgG or an anti-NS5A MAb (Fig. 7D, upper panels) or an anti-NS4B Ab (data not shown). In contrast, only E2 protein, but not CD81, was detected in the high-density pool (Fig. 7D, lower panels). The association of HCV envelope proteins with exosomes has previously been reported [49]. Following the treatment of virus samples with detergent (0.01% saponin), the virion core of both the low- and high-density pools was stained with anti-Core MAb (Fig. 7E). Interestingly, the virion core of LDF, but not the HDF, could be stained with anti-NS5A antibody, but neither normal IgG nor anti-NS4B antibody (Fig. 7E). In parallel, the saponin-treated virus of LDF could be stained with both Core and NS5A simultaneously (Fig. 7F). The specificity of staining was confirmed by staining under various treatment conditions (Fig. 7H). In all virus samples, normal IgG or anti-NS4B antibody yielded only background staining. In HDF, anti-CD81 and -NS5A antibodies also yielded very little staining with or without the saponin treatment. In contrast, in LDF, anti-CD81 (40%) and -E2 (25%) stained intact virus samples strongly, while anti-Core (70%) and -NS5A (40%) stained saponin-treated samples strongly.

**Figure 8:**
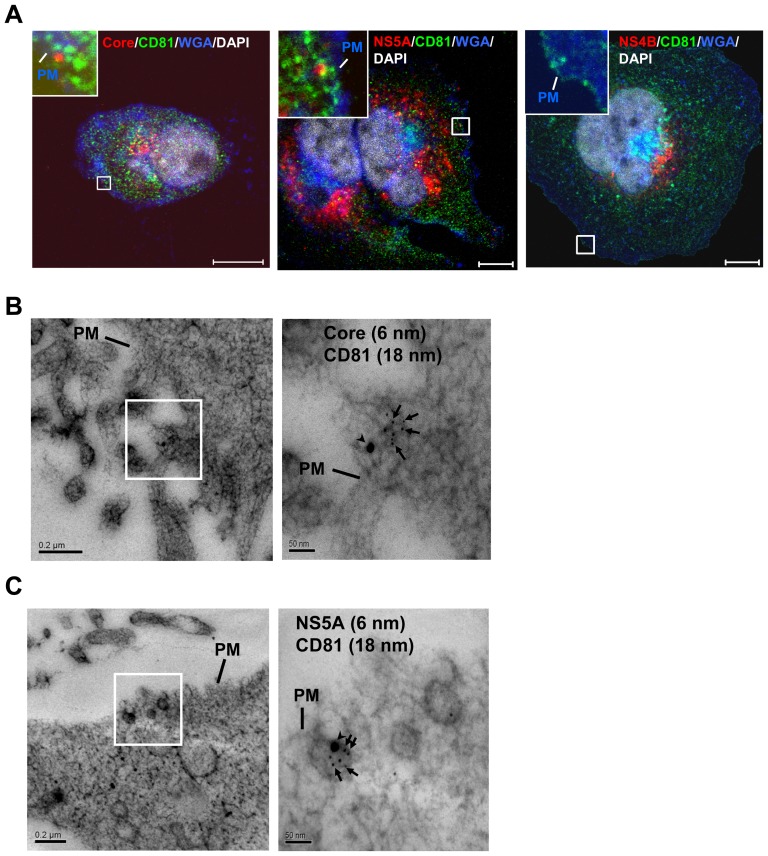
Colocalization of NS5A and Core protein with exosomal proteins at the plasma membrane. (A) The HCV-infected cells (at day 10 p.i.) were co-stained with anti-Core (red) (left panel) or -NS5A (red) (middle panel) or -NS4B (red) (right panel) and anti-CD81 (green). Plasma membrane and nuclei were stained with WGA Alexa Fluor 647 conjugate (blue) and DAPI (gray), respectively. Enlarged views of parts of every image (insets) are shown. PM, plasma membrane; Bars, 10 µm. (B, C) Immuno-EM of plasma membrane co-labeled with antibodies against Core (6 nm) (B) or NS5A (6 nm) (C) and CD81 (18 nm) are shown. Arrowheads, gold-labeled CD81. Arrows, gold-labeled Core and NS5A. Bars, 200 nm and 50 nm (B and C, left and right panels, respectively).

The above results prompted us to further characterize the localization of the exosomal proteins relative to NS5A and Core protein on the plasma membrane. The results indicated that NS5A or Core protein colocalized with CD81 at the plasma membrane by immunofluorescence staining (Fig. 8A) and immuno-EM (Fig. 8B and 8C), but not NS4B. These results again suggest that some NS5A-containing HCV particles exit the cells via fusion of MVB with the plasma membrane. Taken together, these data suggest that a minor population of HCV virion exits cells via exosomes, as NS5A-containing, low-density particles, but the majority of virions do not contain NS5A and exit cells through an exosome-independent pathway.

## Discussion

In this study, we found that in the presence of a microtubule-disrupting drug, both NS5A and Core proteins failed to be transported to the LD (Fig. 2), resulting in the accumulation of NS5A and Core protein in the ER (Fig. 3). In addition, NS5A and Core protein colocalized with microtubule throughout the entire cytoplasm, mostly also associated with LDs (Fig. 1) and at the microtubule end near the plasma membrane (Fig. 6A). These results suggest that the intact microtubule network served as a transport highway for the movement of HCV NS5A (or viral RCs) and Core proteins from the ER towards the LD and plasma membrane for virus assembly and exit. Additionally, it has been shown that the interaction between NS5A and Core protein on LDs plays an important role for virus assembly [18], [19], and that Core protein induces redistribution of LD to the perinuclear region for virus assembly in a microtubule-dependent manner [11]. These studies explain previous finding that the extracellular virus production was decreased by nocodazole [10].

By immunofluorescence staining and EM, we found that NS5A is closely associated with Core protein in almost every step of HCV life cycle, whereas NS4B is not involved in the late steps. However, only a small number (5 signals/cell) of the NS5A-Core complexes were detected at the plasma membrane of the cells, in contrast to Core protein alone (∼63 signals/cell) (Fig. 6C). This finding may explain why the amount of HCV RNA in LDF (∼1×10^4^ copies/ml) was significantly lower than that of HDF (1.3×10^5^ copies/ml) (Fig. 7A). We suggest that NS5A-containing, low-density HCV particles constitute a minor population of the released HCV virions. Nonetheless, this population was apparently infectious, though with slightly lower specific infectivity than the bulk of virus particles in HDF (Fig. 7B). The result is similar to a published report, which showed that less than 2% of all infectivity was in the low-density pool (density of <1.1g/ml) [20]. However, we cannot exclude the possibility that LDF with very weak infectivity may have come from virus contamination from HDF.

Notably, we demonstrated that low-density HCV particles utilize a host exosome biogenesis pathway for the production of NS5A-containing virions. The low-density HCV particles (density of 1.08 g/ml) were also found in hepatitis C patients' serum [26], [52], [53]. Most importantly, NS5A and exosomal markers coincided with Core protein in the low-density fractions (Fig. 7C), which also contained HCV RNA (Fig. 7A). NS5A-Core complexes (Fig. 7F) and exosomal markers (Fig. 7D) were detected in purified LDF virions. Correspondingly, exosomal markers and NS5A or Core protein were colocalized at the plasma membrane, where virus release occurs, as revealed by immunofluorescence staining and Immuno-EM (Fig. 8). Taken together, the results indicated that the low-density, NS5A-containing virions are released off the cell via exosomes, whereas the high-density particles exit the cell via some alternative mechanism not involving the exosomal pathway. Consistent with this hypothesis, HCV particles have been found to be associated with circulating exosomes in serum of HCV-infected patients [49]. Combined with our previous results [10] showing that HCV particles are transported from early to late endosomes/MVB, our current studies suggest that the NS5A-containing HCV particles most likely exit the cells via fusion of MVB to the plasma membrane. Exosomes have been demonstrated to facilitate the budding of human immunodeficiency virus (HIV) [54] and hepatitis A virus (HAV) [55]. Exosome have been known to play important roles in intercellular communications. In HIV and HAV infection, exosomes are required for trans-infection of CD4^+^ T cells [56] and are likely to promote virus spread within the liver [55], respectively. Similarly, exosome-containing HCV virions might fuse to uninfected cell in a mechanism independent of viral envelope proteins and may contribute to HCV natural infection.

Interaction of NS5A with Core protein plays an important role in HCV particle production [19], but its mechanism of involvement in infectious virus production is still unclear. The association of viral NS proteins with viral genomic RNA and their incorporation into viral particles is common among RNA viruses, such as the NS2 of influenza virus [57], the V protein of simian virus 5 [58], the NS3 of coronavirus [59], and the Vpx (viral protein X) of HIV-1 [60]. However, in contrast to our observation, Merz et al. [24] could not observe any NS5A in the virus preparation after affinity purification. The reason for this discrepancy may lie in the sensitivity of detection methods and the method of virus purification, as NS5A was detected only in a minor population of low-density particles in our study. It was also possible that the affinity tag used in the previous study [24] could not be detected in the low-density HCV particles.

Such virion-associated “nonstructural proteins” probably do not play structural roles in virus particles, but may participate in some yet unknown functions required at different steps of the viral life cycle. Recently, an amphipathic α-helical peptide (AH) containing the N-terminal 31 amino acids of NS5A was shown to induce swelling of the vesicles leading to vesicle lysis [61]; in addition, the infectivity of HCV particle was reduced when HCV virions were first treated with the AH peptide, causing the disruption of HCV envelope [62]. Taken together, the data presented here and elsewhere show that NS5A may induce fusion and lysis of lipid vesicles as well as virus particles during the virus entry step. Since NS5A is present inside the virus particles, it is likely that these membrane-altering functions of NS5A could function at a post-binding step, namely, after the virus envelope has been disrupted. Indeed, a postbinding fusion step within an acidic endosomal compartment is required for HCV entry [63]. This will be an interesting topic to study in the future.

In summary, our data demonstrated that a minor population of low-density HCV virions was released in the form of exosome from the infected cells, and that its virion core contains NS5A via close interaction with Core protein during intracellular transport. Our studies provide an additional target for designing new therapeutic approaches by possible use of exosome inhibitor to block the spread of HCV infection.

## Supporting Information

Figure S1 **The HCV-infected cells (at day 10 p.i.) were fixed and processed for EM.** At the right is an enlarged area. The large-diameter (A, white arrows) and small-diameter (B, black arrows) HCV-like particles were released from plasma membrane. PM, plasma membrane; Bars, 500 nm (left panels) and 100 nm (right panels).
(TIF)
