# Supplementary figures and images for: Nonstructural Protein 5A Is Incorporated into Hepatitis C Virus Low-Density Particle through Interaction with Core Protein and Microtubules during Intracellular Transport
Source: PLoS One. 2014 Jun 6;9(6):e99022. doi: 10.1371/journal.pone.0099022 (PMC4048239; doi:10.1371/journal.pone.0099022)

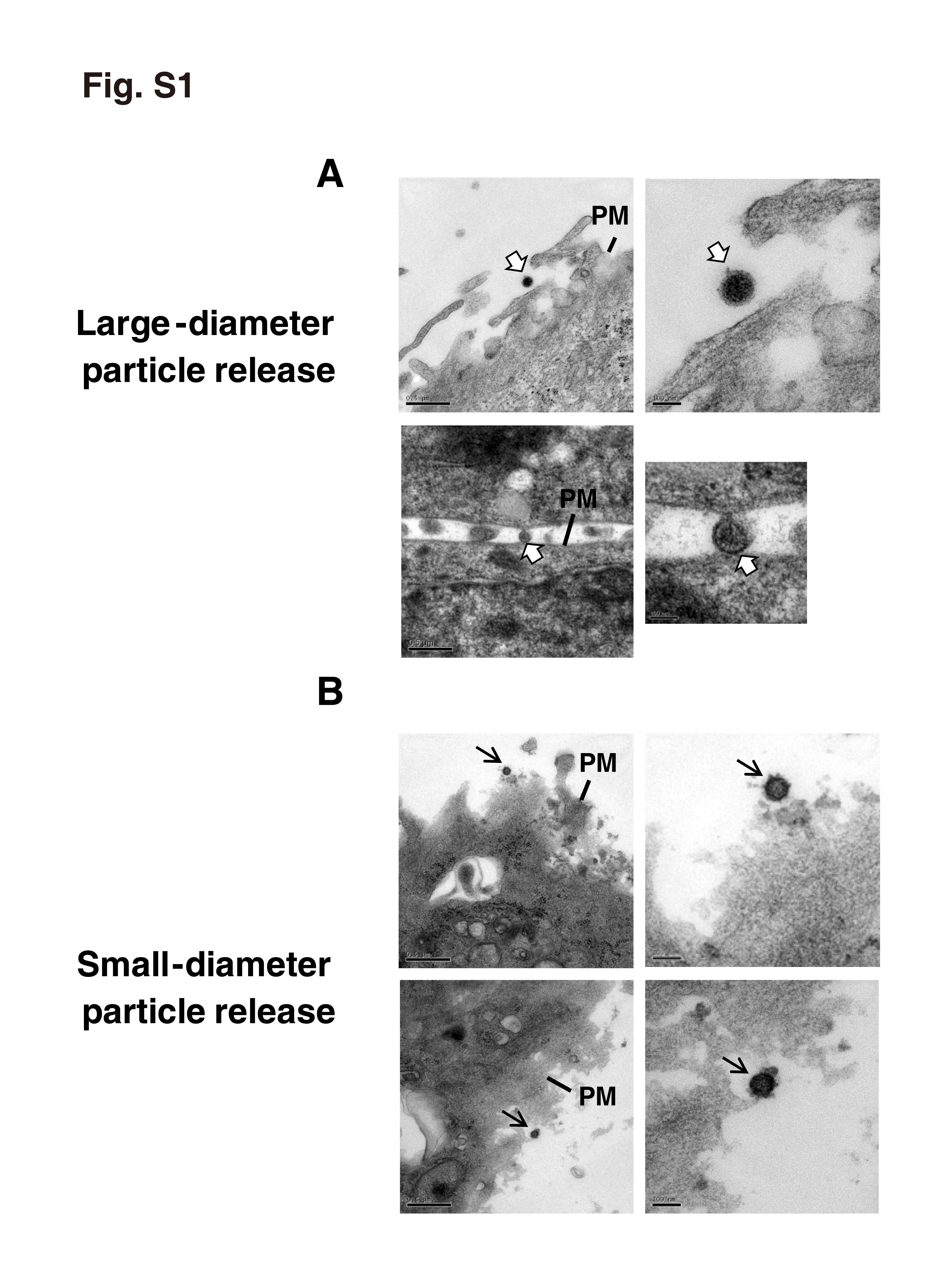

Supplement: Figure S1 — The HCV-infected cells (at day 10 p.i.) were fixed and processed for EM. At the right is an enlarged area. The large-diameter (A, white arrows) and small-diameter (B, black arrows) HCV-like particles were released from plasma membrane. PM, plasma membrane; Bars, 500 nm (left panels) and 100 nm (right panels). (TIF) [file pone.0099022.s001.tif]
